# Codeine is associated with poor prognosis in acute stroke

**DOI:** 10.1002/brb3.869

**Published:** 2017-11-19

**Authors:** Stian T. Ryste, Bernt A. Engelsen, Halvor Naess

**Affiliations:** ^1^ Department of Clinical Science University of Bergen Bergen Norway; ^2^ Institute of Clinical Medicine University of Bergen Bergen Norway; ^3^ Department of Neurology Haukeland University Hospital Bergen Norway; ^4^ Centre for Age‐Related Medicine Stavanger University Hospital Bergen Norway

**Keywords:** acute stroke, cerebrovascular diseases, codeine, pain, prognosis

## Abstract

**Background:**

The aim of this study was to investigate how the use of analgesics, sleeping drugs, and sedatives relates to prognosis and complications in stroke patients in the acute care phase (≤48 hr) after a stroke.

**Materials and Methods:**

Patients with ischemic stroke, hemorrhagic stroke, and transient ischemic attack were included. The study is based on gathering of data on medication from 921 patient records belonging to patients included in the Bergen NORSTROKE registry, 12.2009‐02.2012. In this database risk factors, stroke severity, etiology, and blood analyses were prospectively registered. We have retrospectively registered if patients received one drug or more from a list of analgesics, sleeping drugs, and sedatives within the first 48 hr after admission.

**Results:**

In total, 921 patients were included in the study, 408 females and 513 males. Mean age was 71.0 years. In total, 101 patients were given sleeping drugs, 97 patients sedatives and 140 patients analgesics. Of the group given analgesics, 90 patients were given codeine‐containing analgesics. Logistic regression analyses showed that codeine‐containing analgesics were associated with an increased occurrence of pneumonia (OR = 3.8, *p* < .001), stroke worsening (OR = 2.7, *p* = .001), and a higher mRS‐score (OR = 2, *p* = .024) day 7. The study did not show any relation between poorer prognosis or increased occurrence of complications and the use of other analgesics, sedatives and/or sleeping drugs.

**Conclusion:**

Use of codeine‐containing analgesics is associated with a poorer short‐term prognosis and an increased occurrence of complications in the acute phase after a stroke. The highly significant findings suggest that codeine has a negative effect on acute stroke patients. The study reflects exploratory analyses and prospective studies are necessary to determine the background of the association observed in our study.

## 1 INTRODUCTION

In Norway, an estimated 15,000 cases of stroke occur every year, and the median age is approximately 75 years (Hunskår, 2013). The progressive growth of the elderly population will cause a 50% increase in stroke cases by 2030, provided the incidence remains unchanged (Helsetilsyn, 1999). The majority survives their first stroke, and with intensive care in stroke units the acute mortality is now less than 10% (Fjaertoft & Indredavik, 2007).

Complications in the acute phase include, among others, increased intracranial pressure, epileptic seizures, aspiration pneumonia, urinary tract infection (UTI), urinary retention, deep vein thrombosis (DVT), pulmonary embolism (PE), and muscle and joint pain (National guidelines for treatment and rehabilitation for ischemic stroke, 2010). Complications are associated with poor prognosis. More knowledge is required to determine why acute stroke patients develop complications and thus which complications we can influence and possibly prevent. Acute stroke patients often experience restlessness, sleeping difficulties, and pain. Analgesics, sleeping drugs and sedatives are frequently given and there is limited knowledge of their effect on prognosis and complications. Also, there are very few studies done with respect to the use of opioids in acute stroke. We hypothesized that the sedative effect of these drugs may cause reduced alertness and consciousness, which would make patients more susceptible for complications and less receptive for early rehabilitation.

The aim of this study was to investigate how the use of analgesics, sleeping drugs and sedatives relates to prognosis and accompanying complications in the treatment of stroke patients in the acute care phase (≤ 48 hr) after a stroke.

## 2 MATERIALS AND METHODS

The study is based on gathering of data from 921 patient records belonging patients from Bergen NORSTROKE registry, admitted 12.2009‐02.2012. The Bergen NORSTROKE registry is an ongoing study where all acute stroke patients admitted to the Neurovascular Centre, Department of Neurology, Haukeland University Hospital are included and were relevant background and clinical information is prospectively registered. This includes a National Institute of Health Stroke Scale (NIHSS) score, blood pressure, body temperature and glucose level on admission, patient background including stroke risk factors (hypertension, diabetes, atrial fibrillation, and smoking), dependence prior to stroke (defined as home nursing patients or nursing home patients) and previous illness. The Trial of Org 10172 in Acute Stroke Treatment (TOAST) criteria is used to determine stroke etiology, and the following complications are registered; deep vein thrombosis (DVT), acute myocardial infarction, recurrent stroke, pneumonia, urinary tract infection (UTI), epileptic seizures, and stroke worsening (defined as an increased NIHSS‐score with ≥4 points during the first 72 hr after stroke onset) (Naess, Waje‐Andreassen, Brogger, & Thomassen, 2011).

Patients with ischemic stroke, hemorrhagic stroke and transient ischemic attack (TIA) were included in the study. Subarachnoid hemorrhage patients were not included. The severity of the stroke was assessed by the use of NIHSS‐score on admission, which ranges from 0 to 42 where higher scores indicate greater severity. The patient's level of function was determined by the modified Rankin Scale (mRS) score 7 days after stroke onset (or on discharge if the patient was discharged earlier than after 7 days), where the scores indicate; 0–2 independence, 3–5 dependence and 6 dead. The mRS‐score 7 days after stroke onset is also an indicator of short‐term outcome.

In this study, we retrospectively registered if patients received one drug or more from a list of analgesics, sleeping drugs, and sedatives (Table 1) given within the first 48 hr after admission. The choice of treatment for agitation and pain is based on case‐by‐case evaluation done by the neurologist in charge.

**Table 1:** Classification of the drugs registered in the study

Analgesics	Sleeping drugs	Sedatives
Morphine/ketobemidone	Zopiklone	Diazepam
Codeine‐containing analgesics	Nitrazepam	Haloperidol
Oxycodone	Zolpidem	Levomepromazin
Buprenorphine		Quetiapine
Tramadol		Oxazepam
Fentanyl		Clonazepam

The general standard of care is that patients with minor pain receive paracetamole (acetaminophen), those with moderate pain receive acetaminophen + codeine (hereafter referred to as “codeine‐containing analgesics”) and those with severe pain receive opioids subcutaneously. In regards to agitation haloperidol is often used, levomepromazin if sedation also is desirable, and in milder cases benzodiazepines would be the drug of choice. All analgesics, sleeping drugs, and sedatives given on a routine basis in our stroke unit were included. Opioids were mostly given to patients with severe strokes where life‐sustaining treatment was withdrawn. Therefore, we analyzed opioids in a separate group, but did not expect to find any influence of opioid on outcome because of the very poor prognosis due to stroke severity in the group receiving opioids. A post hoc investigation in the patient records, including nurse charts, why patients received codeine‐containing analgesics was performed.

### 2.1 Statistical analysis

The statistical software STATA 11.0 (http://scicrunch.org/resolver/SCR_012763) was used for the statistical analyses. Student's *t* test and χ^2^ analyses were used for the univariate analyses when appropriate. Logistic regression with different dependent variables (pneumonia or not, stroke worsening or not and higher mRS‐score or not) was used, adjusted for relevant clinical variables.

## 3 RESULTS

In total, 921 patients were included in the study, 408 females and 513 males. There were 758 ischemic strokes, 92 hemorrhagic strokes and 71 TIA. Mean age was 71.0 years (*SD* 15 years). In total, 101 patients (11%) were given sleeping drugs, 97 patients (10.5%) sedatives (simultaneously not given sleeping drugs) and 140 patients (15%) analgesics. Table 2 shows demographics of patients given codeine‐containing analgesics (90 patients) and patients not given codeine‐containing analgesics (831 patients). There was no significant age difference between the two groups, but codeine patients were more often women, they had a higher NIHSS‐score, both on admission and 7 days after stroke onset, and a higher mRS‐score (all *p* < .001). They also had significantly increased mortality (*p* = .021) and occurrence of complications: pneumonia (*p* < .001), UTI (*p* = .04) and stroke worsening (*p* < .001). Codeine‐patients were more often dependent (*p* = .011) and depression was more frequent (*p* = .042).

**Table 2:** Demographics of patients given codeine‐containing analgesics and patients not given codeine‐containing analgesics

	Codeine	Not‐codeine	*p*‐value
Total	90	831	
Male	36 (0.4)	477 (57.4)	.002
Female	54 (0.6)	354 (42.6)	
Age	72.1 (14.7)	70.9 (14.9)	.45
NIHSS on admission	6 (2–14.5)	3 (1–8)	<.001
NIHSS day 7	5 (1–14)	2 (0–6)	<.001
mRS‐score	3 (2–4)	2 (1–4)	<.001
No. of deaths after 90 days	17 (18.9)	89 (10.7)	.021
Dependence prior stroke	20 (22.2)	104 (12.5)	.011
Complications			
Pneumonia	21 (23.3)	66 (7.9)	<.001
Urinary retention	46 (51.1)	226 (27.2)	<.001
Urinary incontinence	28 (31.1)	142 (17.1)	.001
Urinary tract infection	20 (22.2)	96 (11.6)	.004
Stroke worsening	21 (32.3)	91 (14.4)	<.001
Previous illness			
Previous stroke	19 (21.1)	142 (17.1)	.37
Myocardial infarction	13 (14.4)	135 (16.2)	.66
Hypertension	51 (56.7)	434 (52.2)	.42
Diabetes mellitus	10 (11.1)	116 (14.0)	.47
Atrial fibrillation	24 (26.7)	240 (28.9)	.66
Depression	17 (18.9)	116 (14.0)	.042

Table 3 shows the results of logistic regression with pneumonia, stroke worsening and mRS‐score day 7 used as dependent variables. Occurrence of pneumonia was associated with codeine (OR = 3.8, *p* < .001) when adjusted for sex, age, NIHSS on admission, and dependence prior to stroke. Stroke worsening (OR = 2.7, *p* = .001) and higher mRS‐score (OR = 2, *p* = .024) was also associated with the use of codeine. Neither excluding TIA patients nor adjusting for general comorbidity (diabetes mellitus, atrial fibrillation, prior myocardial infarction, and hypertension) changed the results as to the significant relation between the use of codeine and pneumonia, stroke worsening and higher mRS‐score observed in Table 3. The association between codeine and higher occurrence of UTI, urinary retention and urinary incontinence from Table 2 was no longer observed after use of logistic regression. Furthermore, we conducted a subanalysis where only patients given codeine due to headache were included. Out of the 90 codeine patients, 42 were given codeine due to headache and the remaining patients for other reasons, including pain in shoulder, neck, back and hip/pelvis (23 patients), legs and arms (8 patients), and other (17 patients). Even if we only included those who were given codeine due to headache, logistic regression, using the same dependent variables as before, showed pneumonia, stroke worsening, and a higher mRS‐score to be significantly associated with the use of codeine‐containing analgesics.

**Table 3:** The results of logistic regression with pneumonia, stroke worsening and mRS‐score day 7 used as dependent variables

	Pneumonia or not^a^		Stroke worsening or not^b^		mRS 0–2 versus 3–6 day 7^c^	
	Odds ratio (OR)	*p*‐value	Odds ratio (OR)	*p*‐value	Odds ratio (OR)	*p*‐value
Male	.37	<.001	.9	.74	1.1	.72
Age	1.06	<.001	1.03	.001	1.03	<.001
NIHSS on admission	1.12	<.001	1.02	.17	1.3	<.001
Codeine	3.8	<.001	2.7	.001	2	.024
Dependence prior to stroke	.6	.05	‐	‐	5.1	<.001

a Pseudo‐R2 = .2.

b Pseudo‐R2 = .05.

c Pseudo‐R2 = .4.

The study did not show any association between the use of other analgesics, sedatives and/or sleeping drugs, and poorer prognosis or increased occurrence of complications, except for opioids that were almost significantly associated with a higher mRS‐score (*p* = .06).

## 4 DISCUSSION

The results from this study indicate that the use of codeine‐containing analgesics is associated with a poorer short‐term prognosis and an increased occurrence of complications in the acute phase after a stroke. There are potentially several mechanisms that can explain this association. Codeine is a cough suppressant and it is possible that this effect increases the risk of pneumonia. It is also conceivable that codeine contributes to poorer mobilization caused by its sedative effect and nausea, often most pronounced when the patient is in motion (Norsk legemiddelhåndbok, 2016). Early mobilization and functional training related to activities of daily life is one of the key pillars in stroke rehabilitation, and delayed rehabilitation is unfortunate in regards to both prognosis and prevention of complications (Thommessen & Wyller, 2007). It cannot be ruled out that codeine has a direct inhibitory effect on the brain, which is undesirable in early rehabilitation of stroke patients. Stated in another way, it is possible that codeine has direct effects that are unfavorable for acute stroke patients.

The study has not registered when the complications and the neurological worsening arose in the process after the stroke. Thus, we cannot determine whether codeine‐containing analgesics is a direct cause of worsening, or if the patients were given codeine as a consequence of the worsening. For instance, codeine may be given to patients as a result of pain related to stroke worsening or discomfort when developing pneumonia. However, when we conducted a subanalysis where only patients given codeine due to headache were included, the codeine patients still had a significantly higher occurrence of pneumonia, neurological worsening and higher mRS‐score, and it is difficult to believe that headache is associated with poorer prognosis and increased occurrence of complications. Headache is a side effect of the drug dipyridamole. However, there is no reason to believe that dipyridamole is a cause of poorer prognosis and increased occurrence of complications since no such correlation has been found in previous studies (Sacco et al., 2008).

We cannot rule out an unknown factor that explains the association between codeine and increased occurrence of complications and poorer prognosis. However, the highly significant findings imply that there is an association and a direct effect caused by codeine is possible. Codeine has a low affinity for opiate receptors. Thus, the analgesic effect of codeine is mainly caused by O‐detmethylation to morphine, catalyzed by CYP2D6. It has been discussed whether use of codeine in addition to acetaminophen is beneficial for the patients. Around 7%–10% of the population will not, because of dysfunctional expression of CYP2D6, get an analgesic effect from codeine, however, the side effects may still be present. Meta‐analysis show limited effect of codeine used as monotherapy, and the therapeutic effect of adding codeine to acetaminophen in acute pain management is modest (Helland, Spigset, & Slordal, 2004).

As before mentioned there are few studies present in the literature arguing for and against the use of opioids in acute stroke. A cohort study by Juneja et al. investigated the effect of opioids when used as domiciliary treatment in the peri‐stroke period, which is a know practice in the Northern regions of India. Based on mean NIHSS and mRS scores at admission and discharge they found no statistically significant difference, implying that opioid administration neither improved the outcome nor decreased the severity of the stroke (Juneja, Gupta, Singla, Singh, & Kaushal, 2015).

Limitations in our study include the retrospective design as to when and why drugs were given. Also, the indication “headache” found in nurse charts is often based on subjective considerations and potentially a collective term for diffuse complaints. Our study could only partially adjust for prestroke disability and comorbidities. Also, the pseudo‐R2 values for each dependent variable in Table 3 indicate that most of the variance is related to other, unmeasured factors. Another limitation concerns evaluation of opioids that were mostly given to patients with severe strokes where life‐sustaining treatment was withdrawn. This prevents any conclusion as to the effect of opioids on stroke outcome. It is possible that use of opioids could have similar findings to those observed with the use of codeine‐containing analgesics. However, strengths in our study are that the remaining variables from the Bergen NORSTROKE register were prospectively registered.

## 5 CONCLUSION

Results from this study conclude that use of codeine‐containing analgesics is associated with a poorer short‐term prognosis and an increased occurrence of complications in the acute phase after a stroke. Using the same conditions, there were no similar findings in the use of other analgesics, sedatives and/or sleeping drugs. The highly significant findings give rise to suspicion that codeine has a negative effect on acute stroke patients. However, our study reflects exploratory analyses and prospective studies are necessary to determine the background of the association observed in our study.
